# An Organoid Model for the Therapeutic Effect of Hyperthermic Intraperitoneal Chemotherapy for Colorectal Cancer

**DOI:** 10.1245/s10434-024-16469-1

**Published:** 2024-11-26

**Authors:** Duo Liu, Zexin Chen, Weihao Deng, Jianqiang Lan, Yu Zhu, Huaiming Wang, Xing Xu, Yuanxin Zhang, Xiangwei Wu, Keli Yang, Jian Cai

**Affiliations:** 1 Department of Colorectal Surgery, Shenzhen Second People’s Hospital, First Affiliated Hospital of Shenzhen University, Medical Innovation Technology Transformation Center of Shenzhen Second People’s Hospital, Shenzhen University, Shenzhen, China; 2 Guangdong Research Center of Organoid Engineering and Technology, Accurate International Biotechnology Co. Ltd., Guangzhou, China; 3 Department of Pathology, Guangdong Institute of Gastroenterology, Guangdong Provincial Key Laboratory of Colorectal and Pelvic Floor Diseases, Biomedical Innovation Center, The Sixth Affiliated Hospital, Sun Yat-Sen University, Guangzhou, China; 4 Department of Colorectal Surgery, Guangdong Institute of Gastroenterology, Guangdong Provincial Key Laboratory of Colorectal and Pelvic Floor Diseases, Biomedical Innovation Center, The Sixth Affiliated Hospital, Sun Yat-Sen University, Guangzhou, China; 5 Department of Breast and Thyroid Surgery, Shenzhen Second People’s Hospital, First Affiliated Hospital of Shenzhen University, Medical Innovation Technology Transformation Center of Shenzhen Second People’s Hospital, Shenzhen University, Shenzhen, China; 6 Qiantang Biotechnology Co. Ltd., Suzhou, China

**Keywords:** Colorectal cancer, Peritoneal metastasis, Hyperthermic intraperitoneal chemotherapy, Organoids

## Abstract

**Background:**

Consensus regarding the hyperthermic intraperitoneal chemotherapy (HIPEC) for colorectal cancer (CRC) regimen remains elusive. In this study, patient-derived tumor organoids from CRC were utilized as a preclinical model for in vitro drug testing of HIPEC regimens commonly used in clinical practice. This approach was used to facilitate the clinical formulation of HIPEC.

**Method:**

Tumor tissues and corresponding clinical data were obtained from patients diagnosed with CRC at the Sixth Affiliated Hospital of Sun Yat-Sen University. Qualified samples were cultured and passaged. We aimed to assess the sensitivity of in vitro hyperthermic perfusion using five different regimens, i.e. mitomycin C, mitomycin C combined with cisplatin, mitomycin C combined with 5-fluorouracil, oxaliplatin, and oxaliplatin combined with 5-fluorouracil.

**Results:**

Tumor organoids obtained from 46 patients with CRC were cultured, and in vitro hyperthermic perfusion experiments were conducted on 42 organoids using five different regimens. The average inhibition rate of mitomycin C was 85.2% (95% confidence interval [CI] 80.4–89.9%), mitomycin C combined with cisplatin was 85.5% (95% CI 80.2–90.7%), mitomycin C combined with 5-fluorouracil was 65.6% (95% CI 59.6–71.6%), oxaliplatin was 37.9% (95% CI 31.5–44.3%), and oxaliplatin combined with 5-fluorouracil was 40.7% (95% CI 33.9–47.5%).

**Conclusion:**

In vitro hyperthermic perfusion demonstrates that the inhibition rate of mitomycin C, both alone and in combination with cisplatin, surpasses that of the combination of mitomycin C with 5-fluorouracil and oxaliplatin. In clinical practice, the combination of mitomycin C and cisplatin can be regarded as the optimal choice for HIPEC in CRC.

Colorectal cancer (CRC) ranks as the third most prevalent malignancy globally, with the second highest fatality rate.^1^ Peritoneal metastasis (PM) manifests in around 10% of individuals diagnosed with CRC.^2^ The prognosis of patients with PM from CRC is poor and the median survival time is only about 6 months.^3^ In the 1990s, Jacquet et al. proposed cytoreductive surgery (CRS) combined with hyperthermic intraperitoneal chemotherapy (HIPEC) for the treatment of PM from CRC,^4^ and this strategy is now widely adopted in many centers around the world.^5^ Several studies have confirmed that CRS+HIPEC can significantly improve the survival of patients with CRC PM who can achieve complete or near-complete resection of PM.^6–8^

Nevertheless, consensus regarding the HIPEC regimen remains elusive, as evidenced by a comprehensive analysis of 171 HIPEC reports. The study reveals the existence of a minimum of 60 distinct HIPEC regimens, predominantly employing oxaliplatin and mitomycin C.^9^ The current retrospective studies have generated conflicting outcomes when investigating the efficacy of oxaliplatin and mitomycin C in HIPEC for CRC.^10,11^ In contrast, the two current prospective studies of HIPEC using oxaliplatin were negative. The PRODIGE7 study showed that oxaliplatin-based HIPEC did not provide a survival benefit,^12^ while the COLOPEC study showed that postoperative prophylactic use of oxaliplatin-based HIPEC in patients with T4 or perforated colon cancer did not reduce the incidence of postoperative PM.^13^ This calls into question the role of the HIPEC regimen with oxaliplatin; however, it is difficult to conduct prospective studies to compare the efficacy of HIPEC between different drugs. Exploration of the efficacy of different HIPEC regimens in vitro may inform clinical decision making.

At present, the commonly used in vitro models mainly include cancer cell lines, patient-derived tumor xenografts (PDTXs), and organoids. Among them, tumor organoids are three-dimensional (3D) cultures of cancer cells that can be obtained from each patient’s tumor cells.^14^ A large amount of evidence has confirmed that there is a high similarity in histopathological molecular characteristics, drug responsiveness, and genetic stability between subject-derived tumor organoids and their original specimens.^15–18^ In contrast to conventional cancer models that require large sample sizes, organoids can be grown from small sample sizes (extracted from needle biopsies), with high success rates for personalized tumor modeling.^19^ In subjects with resected tumors, multiple types of organs can be generated from different regions of the tumor to better mimic tumor heterogeneity. Therefore, tumor organoids as preclinical models for drug sensitivity screening have been gradually used to guide clinical individualized drug use,^20^ an approach that has high accuracy in predicting the treatment efficacy of clinical patients. Vlachogiannis et al. constructed an organoid model of patients with metastatic drug-resistant CRC that achieved 100% sensitivity, 93% specificity, 88% positive predictive value, and 100% negative predictive value in predicting the efficacy of targeted drugs or chemotherapy.^21^

Currently, studies utilizing organoid models to evaluate HIPEC for CRC involve small samples and have limited treatment regimens. Therefore, this study is the first to use CRC patient-derived tumor organoids as a preclinical model to perform in vitro drug testing of four HIPEC regimens commonly used in clinical practice in large samples to assist clinical development of HIPEC treatment.

## Materials and Methods

### Acquisition of Tumor Organoids and Patient Clinical Data

CRC organoids were acquired from patients diagnosed with CRC at the Sixth Affiliated Hospital of Sun Yat-Sen University. Following surgery, a fraction of the tumor tissues that were removed was collected. The clinical data obtained encompassed the following parameters: sex, age, body surface area, primary tumor location, histological characteristics, TNM staging, KRAS gene mutation, BRAF gene mutation, mismatch repair (MMR) protein status, and history of oxaliplatin chemotherapy prior to tissue sampling. The Ethics Committee of the Sixth Affiliated Hospital of Sun Yat-Sen University granted approval for this study (approval number 2022ZSLYEC-446).

### Formulation of the Hyperthermic Intraperitoneal Chemotherapy (HIPEC) Protocol In Vitro

Four HIPEC regimens were selected on the basis of the current study (Table 1).

**Table 1:** Four selected hyperthermic intraperitoneal chemotherapy regimens

Drug	MMC	MMC+CDDP	MMC+5-FU	OXA+5-FU
Concentration	35 mg/m^2^ ^22,23^	MMC 5 mg/L+ CDDP 20 mg/L^24^	MMC 2 mg/L+5-FU 200 mg/L^25^	5-FU 400 mg/m^2^ IV + OXA 460 mg/m^2^ ^22,26,27^
Time	90 min	90 min	30 min	30 min
Temperature	42 °C	42 °C	42 °C	42 °C

*MMC* mitomycin C, *CDDP* cis-platinum, *5-FU* 5-fluorouracil, *OXA* oxaliplatin, *IV* intravenously

The cumulative dosage of mitomycin C administered for intraperitoneal hyperthermic perfusion chemotherapy was 35 mg/m^2^, divided into three doses: an initial dose of 17.5 mg/m^2^, followed by two additional doses of 8.75 mg/m^2^ at 30-min intervals. The objective was to maintain a consistent and stable concentration in the perfusate and abdominal cavity throughout the entire process, with the mitomycin C concentration remaining approximately equal to the initial concentration.^28^ On average, the body surface area of adults falls within the range of 1.5–2.0 m^2^, whereas the volume of the HIPEC solution is 3 L. The administration of oxaliplatin required an intravenous injection of 5-fluorouracil 400 mg/m^2^ given 30 min before the oxaliplatin dose; according to the study, the intraperitoneal concentration of 5-fluorouracil was approximately 5 mg/L given 30 min after the intravenous injection.^29^ To enhance the simulation of hyperthermic intraperitoneal perfusion of oxaliplatin in clinical settings, the addition of 5-fluorouracil (5 mg/L) was compared with oxaliplatin alone. Consequently, the HIPEC protocol implemented for experimentation in this study is illustrated in Table 2.

**Table 2:** Experimental protocol used for in vitro hyperthermic perfusion in this study

Drug	MMC	MMC + CDDP	MMC + 5-FU	OXA	OXA + 5-FU
Concentration	10 mg/L (30 μmol/L)	5 mg/L + 20 mg/L (15 μmol/L + 67 μmol/L)	2 mg/L + 200 mg/L (6 μmol/L + 1.5 mmol/L)	230 mg/L (579 μmol/L)	230 mg/L + 5 mg/L (579 μmol/L + 38 μmol/L)
Time	90 min	90 min	30 min	30 min	30 min
Temperature	42 °C	42 °C	42 °C	42 °C	42 °C

*MMC* mitomycin C, *CDDP* cis-platinum, *5-FU* 5-fluorouracil, *OXA* oxaliplatin

### Organoid Culture and Passage

After washing the samples, the tissue was cut into small pieces measuring approximately 1 mm^3^ using scissors. ‘Digest solution I’ (DMEM/F12, supplemented with type IV collagenase and hyaluronidase) was then added to the tissue block and shaken in a shaker for 2–4 h at 37°C. The precipitate was centrifuged at 1300 rpm for 3 min, and ‘digest solution II’ (DMEM/F12, supplemented with dispase and DNAse) was added to the precipitate and digested in a shaker at 37°C for 10–20 min. The precipitate was reconstituted in Hank’s balanced salt solution. The filtrate was then obtained through filtration using a 70 μm cell screen and subjected to centrifugation. Subsequently, the cell precipitate was resuspended in a medium and the resulting cell suspension was blended with matrigel in the desired proportion. Approximately 50–60 μL of the combined cell liquid was carefully dispensed into the Petri dish and the dish was subsequently transferred into a carbon dioxide incubator to allow for solidification. Finally, 4 mL of medium was added. Regular observations of the samples' growth were conducted at intervals of 2–3 days, and the appropriate timing for fluid exchange or passage was determined based on these observations. In the event of a subpar performance in the sample activity, it may be necessary to terminate the culture prematurely and replace it with alternative samples.

### In Vitro Hyperthermic Perfusion Experiments

In adherence with the experimental protocol, arrangement of the organoid drug sensitivity paving plate was intricately planned, with a clear distinction between the experimental and control groups. The experimental group received drug treatment, while the control group did not receive any drugs and was cultured under normal conditions at 37°C.

Once the enrolled samples were observed and documented using a microscope, the culture dish was carefully emptied of its medium, while retaining the adhesive droplets. The culture dish then received an infusion of 3 mL TrypLE Express Enzyme (1X) digestion solution. The expulsion of the gel drops promptly followed, and the larger organoid samples were then positioned in a 37°C incubator for digestion, with each cycle lasting 3–5 min. Upon digestion of the organoid into an homogeneous cell mass comprising multiple cells, termination of the digestion process was achieved by adding a double volume of Advanced DMEM/F12 medium. Following centrifugation, the supernatant was discarded and the cells were resuspended in 200 μL of CRC organoid medium. A 10 μL sample was taken for counting and the corresponding volume of organoids was diluted to a concentration of 2000 cells/well for plating.

A suspension of cells was prepared using CRC organoid culture medium and subsequently diluted in a 2:3 ratio of medium to glue. The adhesive mixture was divided into a 96-well plate, with each well containing 10 μL, using a single-channel pipettor. A mixture of culture medium and gelatin was prepared in a 1:1 ratio, and 10 μL of this mixture was distributed into each well of a 96-well plate to serve as the blank control. Following a 30 min incubation period, the preheated medium was added at a rate of 90 μL per well; 90 μL of normal saline was then dispensed into the empty wells surrounding both the drug test and control wells to seal the edges.

Following a 2 day period of inoculating the organoids into 96-well plates, the incubator was preheated to 42 °C while also preheating the organoid culture medium. The drugs that were diluted underwent preheating in an incubator set at 42 °C. Once the preheating was complete, the drugs were incorporated into the experimental wells. Subsequent to the hot perfusion experiment, the drugs present in the organoid wells were exchanged with medium to enable further cultivation.

### Organoid Activity Test

Cell viability was determined using the adenosine triphosphate (ATP) chemiluminescence assay. Following the application of drug-infused thermoperfusion to the organoids, the condition of each well sample was examined under a microscope. As a result, the medium was aspirated and replaced with 50 μL of cell recovery solution, which was then incubated at 4 °C for 1 h. The addition of 50 μL of luminescent reagent (Promega, Madison, WI, USA) took place before the detection process, and the test was subsequently conducted using the machine.

Calculation of the inhibition ratio was performed as follows: ATP luminescence value of the control group − ATP luminescence value of the experimental group)/ATP luminescence value of the control group * 100%.

### Statistical Analysis

Statistical analysis was conducted using SPSS 26.0 (IBM Corporation, Armonk, NY, USA) and GraphPad Prism 9.0 (GraphPad, San Diego, CA, USA) software. Measurement data that followed a normal distribution were presented as the mean (standard deviation or 95% confidence interval), while data that did not follow a normal distribution were presented as the median (interquartile range). The qualitative data were presented as number of cases (%). To compare measurement data with a normal distribution, the t-test was employed, while the Wilcoxon rank-sum test was utilized in instances where the data did not follow a normal distribution. Furthermore, a comparison of measurement data among multiple groups was performed using the analysis of variance. Statistical significance was determined by considering two-sided *p*-values <0.05.

### RNA-Seq Analysis

The Eastep^®^ Super Total RNA Extraction Kit (Progema, Shanghai, China) was utilized for the extraction of total RNA, following the instructions provided in the reagent manual.

One microgram total RNA was used for following library preparation. Paired-end (PE) libraries were prepared using the VAHTS^®^ Universal V8 RNA-seq Library Prep Kit (Vazyme Technology Co., Ltd, Nanjing, China). Poly(A) mRNA isolation was performed using Oligo(dT) beads, and the mRNA fragmentation was performed using divalent cations and high temperature. Priming was performed using random primers. First- and second-strand complementary DNA (cDNA) were synthesized. The purified double-stranded cDNA was then treated to repair both ends and to add a dA-tailing in one reaction, followed by a T-A ligation to add adaptors to both ends. Size selection of adaptor-ligated DNA was then performed using DNA Clean Beads. Each sample was then amplified by polymerase chain reaction (PCR) using P5 and P7 primers, and the PCR products were validated. Libraries with different indexes were then multiplexed and loaded on a Navoseq6000 instrument for sequencing using a 2×150 PE configuration according to the manufacturer’s instructions.

Sequencing reads that contain low-quality, adaptor-polluted, and high content of unknown base (N) reads should be processed and removed before downstream analyses. We filtered the low-quality reads (more than 20% of the bases’ qualities were lower than 10), reads with adaptors, and reads with unknown bases (N bases >5%) to get the clean reads. We used fastp to filter and Bowtie2 for the mapping step.

We mapped the clean reads to the reference using Bowtie2, and then calculated the gene expression level using RSEM. We then calculated the Pearson correlation between all samples using the cor function, performed hierarchical clustering between all samples using the hclust function, performed PCA analysis with all samples using the princomp function, and drew the diagrams using ggplot2 in R.

We detected different expression genes (DEGs) with DEseq2, as requested. DEseq2 is based on the Poisson distribution. The parameters used were fold change ≥2 and adjusted *p*-values ≤ 0.05.

Using the KEGG annotation result, we classified DEGs according to the official classification, and also performed KEGG functional enrichment using phyper, a function of R. We then calculated the false discovery rate (FDR) for each *p*-value. In general, terms with an FDR no larger than 0.05 were defined as significantly enriched.

## Results

### Patient Information

Cultivation of organoids from tumor tissue derived from 46 CRC patients was successfully achieved. Of the 46 total patients, 29 were male and 17 were female. The mean age was 55.2 years (range 29–83 years) and the mean body surface area was 1.76 (range 1.39–2.07). The data included 8 cases of right hemicolon cancer, 23 cases of left hemicolon cancer, 15 cases of rectum cancer, 40 cases of moderately or well differentiated adenocarcinoma, 4 cases of poorly differentiated adenocarcinoma, 2 cases of mucinous adenocarcinoma, 4 cases of liver metastasis, 4 cases of lung metastasis, 8 cases of PM, and 8 cases of KRAS gene mutation. Among the patients included in the study, one patient exhibited MMR deficiency, while 13 had previously undergone oxaliplatin chemotherapy. The remaining general clinical characteristics are shown in Table 3.

**Table 3:** Clinical characteristics of patients

Characteristics	*n* = 46 (%)
Gender	
Male	29 (63.0)
Female	17 (37.0)
Age	
Mean (SD)	55.2 (13.4)
Body surface area	
Mean (SD)	1.76 (0.17)
Primary tumor site	
Colon	31 (67.4)
Rectum	15 (32.6)
Histologic features	
Moderately to well-differentiated adenocarcinoma	40 (87.0)
Poorly differentiated adenocarcinoma as well as mucinous adenocarcinoma and signet ring cell carcinoma	6 (13.0)
TNM T stage	
Tis-T2	8 (17.4)
T3-4	34 (73.9)
Unknown	4 (8.7)
TNM N stage	
N0	23 (50.0)
N1-2	19 (41.3)
Unknown	4 (8.7)
Hepatic metastases	
Yes	4 (8.7)
No	42 (91.3)
Pulmonary metastasis	
Yes	4 (8.7)
No	42 (91.3)
Peritoneal metastasis	
Yes	8 (17.4)
No	38 (82.6)
KRAS	
Wild	10 (21.7)
Mutation	8 (17.4)
Undetected	28 (60.9)
BRAF	
Wild	18 (39.1)
Mutation	0
Undetected	28 (60.9)
MMR	
pMMR	42 (91.3)
dMMR	1 (2.2)
Undetected	3 (6.5)
Systemic chemotherapy with oxaliplatin was used before sampling	
Yes	13 (28.3)
No	33 (71.7)

*SD* standard deviation, *MMR* mismatch repair, *pMMR* mismatch repair proficient, *dMMR* mismatch repair deficient

### Organoid Culture Results and Identification

**Fig. 1:**
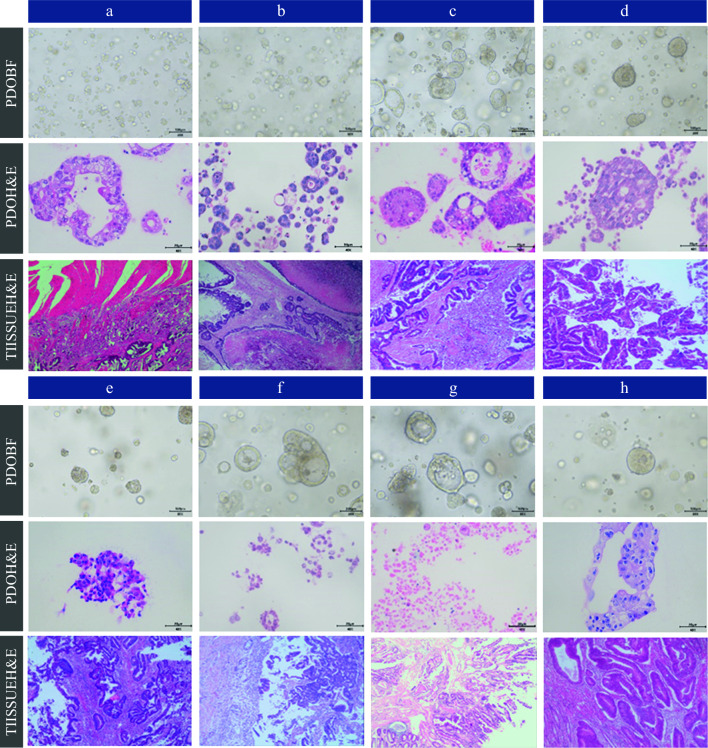
Morphology of organoids grown from tumor tissues of some patients, and H&E staining of organoids and tumor tissues (first row: morphology of organoids (20×), second row: H&E staining of organoids (40×), third row: H&E staining of original tumor tissues). *H&E* hematoxylin and eosin

The organoids derived from the primary tumor tissue or metastatic tissue of 46 patients exhibited distinct morphological characteristics (a selection of which are depicted in Fig. 1). The organoids depicted in Fig. 1 were cultivated from peritoneal metastatic tissue. The organoids depicted in Fig. 1e and h were cultivated from tissue of colon cancer origin, and the organoids depicted in Fig. 1d, f, and g were cultivated from tissue obtained from rectal cancer. The tumor tissue depicted in Fig. 1d was identified as well-differentiated adenocarcinoma, and the tumor tissues displayed in Fig. 1a, b, c, e, f, g, and h exhibited characteristics of moderately differentiated adenocarcinoma. The morphology of organoids primarily exhibited tubular structures (Fig. 1a, f, h) or solid growth (Fig. 1d). Hematoxylin and eosin staining revealed a large number of cancerous cells, closely resembling the histology of the original metastases.

### Establishment of a Hyperthermic Perfusion System In Vitro

**Fig. 2:**
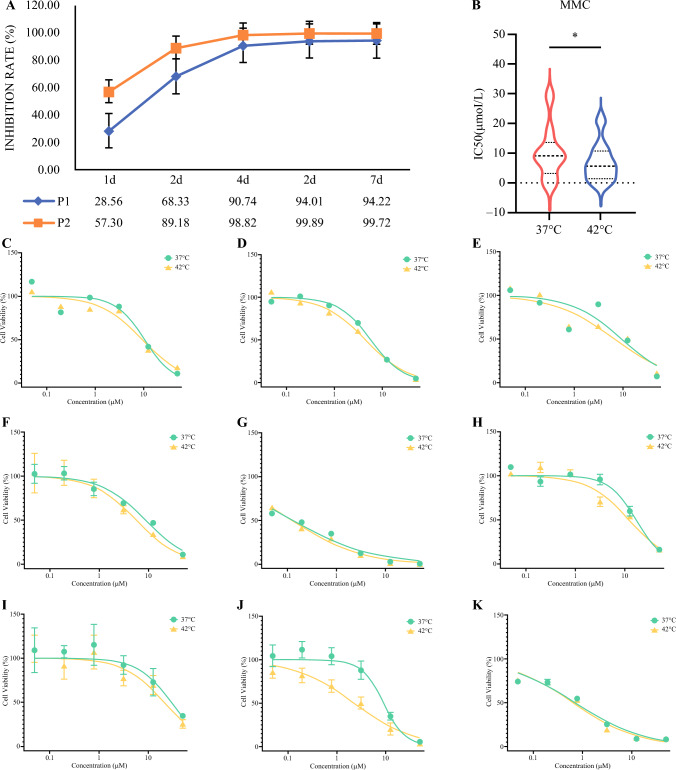
**A** Inhibition rate at different detection times after in vitro hyperthermic perfusion with mitomycin C. **B** IC_50_ distribution of MMC at 37 °C and 42 °C. **C–K** Dose-response curves for MMC (organoids a–i) at 37 °C and 42 °C. *P1* patient-derived organoid 1, *P2* patient-derived organoid 2, *IC*_*50*_ half maximal inhibitory concentration, *MMC* mitomycin C

For the purpose of determining the optimal timing to assess the inhibition rate after hyperthermic perfusion of CRC organoids in vitro, we treated two CRC organoids with mitomycin C (30 μmol/L, 42 °C, 90 min). The determination of the inhibition rate was conducted at specific time intervals, specifically on the first, second, fourth, sixth, and seventh day following treatment. The findings revealed that the peak rate of inhibition was observed approximately 4–6 days after in vitro hyperthermic perfusion of the organoids (Fig. 2a). As a result, the assessment of cell viability in this study was performed on day 4 after the hyperthermic perfusion treatment.

The objective of this study was to assess the drug responsiveness of the organoid hyperthermic perfusion model to mitomycin C hyperthermic perfusion. The in vitro hyperthermic perfusion procedure consisted of treating nine organoids with different concentrations of mitomycin C (50.00 μmol/L, 12.50 μmol/L, 3.13 μmol/L, 0.78 μmol/L, 0.20 μmol/L, and 0.05 μmol/L). Organoids were exposed to mitomycin C for a duration of 90 min at temperatures of 37 °C and 42 °C, and the inhibition rate was calculated on day 4 after drug treatment. The dose-response curves were plotted separately (Fig. 2c–k), indicating significant variations in drug sensitivity among organoids derived from different patients (Table 4). The mean half maximal inhibitory concentration (IC_50_) of mitomycin C at a temperature of 37 °C and 42°C was 10.036 μmol/L (95% CI 3.300–16.773 μmol/L) and 6.930 μmol/L (95% CI 1.902–11.959 μmol/L), respectively (paired t-test *p* = 0.013) (Fig. 2b). The results demonstrate that heating has the potential to increase the cytotoxic effect of mitomycin C, while also confirming the stability of the in vitro hyperthermic perfusion system utilized in this study.

**Table 4:** IC_50_ values of mitomycin C at 37 °C and 42 °C, and inhibition rate at the maximum concentration (9 organoids)

Organoids	37 °C		42 °C	
	IC_50_ (95% CI) [μmol/L]	Inhibition rate of maximum concentration (%)	IC_50_ (95%CI) [μmol/L]	Inhibition rate of maximum concentration (%)
Organoids a	10.530 (4.407–26.740)	89.1	9.497 (5.281–17.40)	82.0
Organoids b	5.786 (4.653–7.191)	95.1	4.457 (3.244–6.108)	95.6
Organoids c	9.427 (1.365–238.500)	92.7	6.767 (1.931–29.810)	89.2
Organoids d	8.728 (6.214–12.180)	89.1	5.688 (3.649–9.124)	95.4
Organoids e	0.134 (0.049–0.271)	99.5	0.129 (0.068–0.213)	99.7
Organoids f	16.760 (14.010–20.210)	83.6	12.060 (8.956–16.320)	83.2
Organoids g	29.090 (17.010–56.690)	65.4	20.870 (12.420–37.300)	74.5
Organoids h	9.161 (7.008–11.780)	94.3	2.264 (1.604–3.168)	96.1
Organoids i	0.713 (0.531–0.948)	91.8	0.647 (0.488–0.852)	91.0
Average	10.036 (3.300–16.773)	89.0 (81.4–96.6)	6.930 (1.902–11.959)	89.6 (83.3–96.0)

*IC*_*50*_ half maximal inhibitory concentration, *CI* confidence interval

### Sensitivity of In Vitro Thermoperfusion of Five Regimens at Clinical Doses

**Fig. 3:**
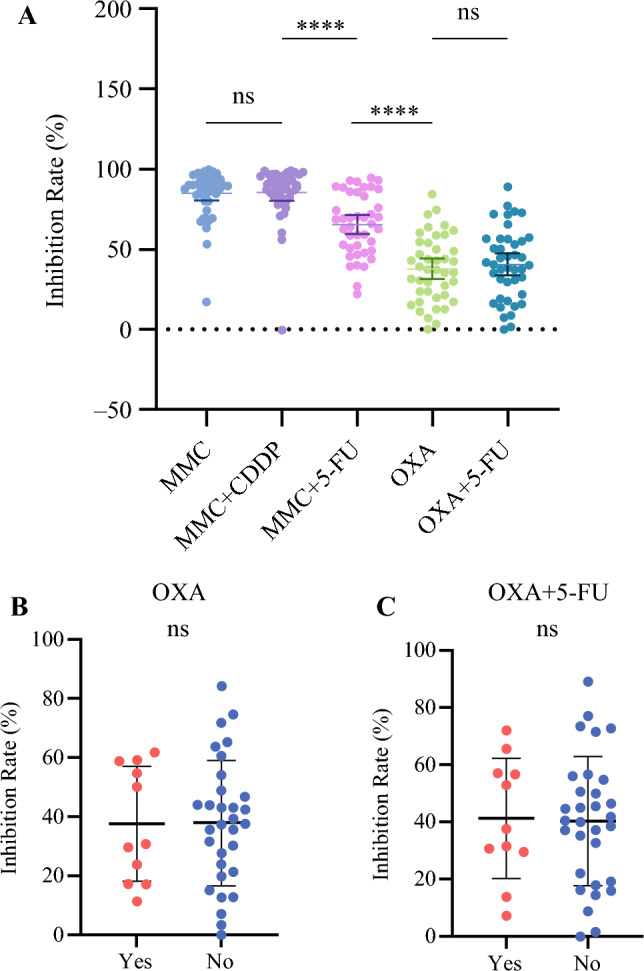
**A** Inhibition rate of five regimens on in vitro hyperthermic perfusion of colorectal cancer organoids at clinical doses. MMC: mitomycin C; CDDP: cis-platinum; 5-FU: 5-fluorouracil; OXA: oxaliplatin. **B** and **C** The inhibition rate of oxaliplatin and oxaliplatin combined with 5-fluorouracil in vitro hyperthermic perfusion with or without oxaliplatin chemotherapy history OXA: oxaliplatin; 5-FU: 5-fluorouracil

In order to evaluate the thermoperfusion sensitivity of five distinct regimens on CRC organoids, namely mitomycin C, mitomycin C combined with cisplatin, mitomycin C combined with 5-fluorouracil, oxaliplatin, and oxaliplatin combined with 5-fluorouracil, a total of 42 organoid cases were employed for clinical dose testing (Table 2). The results indicated that the use of mitomycin C alone resulted in an average inhibition rate of 85.2% (95% CI 80.4–89.9%). Furthermore, the average inhibition rate of 65.6% (95% CI 59.6–71.6%) was observed for the combination of mitomycin C and 5-fluorouracil, whereas oxaliplatin exhibited an average inhibition rate of 37.9% (95% CI 31.5–44.3%). Subsequently, the Dunnett’s T3 test was employed to compare the two groups (Table 5). The findings indicated no statistically significant difference in the inhibition rate between mitomycin C and the combination of mitomycin C and cisplatin (*p *> 0.999). In addition, no statistical disparity was observed in the inhibition rate between oxaliplatin and the combination of oxaliplatin with 5-fluorouracil (*p *> 0.999). The inhibitory rate of the combination of mitomycin C and cisplatin was significantly greater than that of the combination of mitomycin C and 5-fluorouracil (*p *< 0.001), and the inhibitory rate of mitomycin C combined with 5-fluorouracil was superior to that of oxaliplatin combined with 5-fluorouracil (*p *< 0.001) (Fig. 3a).

**Table 5:** The inhibition rate of five protocols for in vitro thermoperfusion of colorectal cancer organoids at clinical dose and the comparison of the two protocols

Thermoperfusion regimen	Average inhibition rate [%] (95% CI)	*p-*Value
MMC	85.2 (0.4–89.9)	
MMC + CDPP	85.5 (80.2–90.7)	>0.999
MMC + 5-FU	65.6 (59.6–71.6)	<0.001
OXA	37.9 (31.5–44.3)	<0.001
OXA + 5-FU	40.7 (33.9–47.5)	<0.001
MMC + CDPP	85.5 (80.2–90.7)	
MMC + 5-FU	65.6 (59.6–71.6)	<0.001
OXA	37.9 (31.5–44.3)	<0.001
OXA + 5-FU	40.7 (33.9–47.5)	<0.001
MMC + 5-FU	65.6 (59.6–71.6)	
OXA	37.9 (31.5–44.3)	<0.001
OXA + 5-FU	40.7 (33.9–47.5)	<0.001
OXA	37.9 (31.5–44.3)	
OXA + 5-FU	40.7 (33.9–47.5)	>0.999

*MMC* mitomycin C, *CDDP* cis-platinum, *5-FU* 5-fluorouracil, *OXA* oxaliplatin, *CI* confidence interval

### Sensitivity of Peritoneal Metastatic versus Primary Tumor-Derived Organoids to Five Regimens of In Vitro Hyperthermic Perfusion at Clinical Doses

To address the issue of the low proportion of PM-derived organoids in this study, we conducted a comparison of the inhibition rate between PM tissue-derived organoids and primary CRC tissue-derived organoids using five in vitro hyperthermic perfusion schemes. A total of 7 cases involved organoids derived from PM tissue, while 35 cases involved organoids derived from primary tumor tissue. According to the results of the independent sample t-test, there was no significant disparity in the inhibition rate of the two organs when subjected to the five variations of in vitro hyperthermic perfusion (Table 6).

**Table 6:** Rates of peritoneal metastasis and primary tumor organoid suppression according to five regimens

Thermoperfusion regimen	Average inhibition rate [%] (95% CI)		*p-*Value
	Organoids of peritoneal metastatic	Organoids of primary tumor	
MMC	84.0 (74.8–93.1)	85.4 (79.9–91.0)	0.822
MMC + CDPP	83.2 (72.0–94.4)	85.9 (79.8–92.0)	0.698
MMC + 5-FU	56.0 (34.7–77.3)	67.5 (61.3–73.8)	0.149
OXA	33.9 (11.7–56.0)	38.7 (31.8–45.6)	0.575
OXA + 5-FU	32.3 (9.4–55.1)	42.4 (35.1–49.7)	0.269

*MMC* mitomycin C, *CDDP* cis-platinum, *5-FU* 5-fluorouracil, *OXA* oxaliplatin, *CI* confidence interval

### Effect of Oxaliplatin Chemotherapy History on Sensitivity to Oxaliplatin Hyperthermic Perfusion In Vitro

For the purpose of further investigating the influence of previous oxaliplatin chemotherapy on the sensitivity of oxaliplatin hyperthermic perfusion in vitro, we conducted a comparative analysis of the inhibition rate of oxaliplatin and oxaliplatin combined with 5-fluorouracil in patients with and without a history of oxaliplatin chemotherapy prior to tumor tissue sampling; 11/42 patients had been treated with a chemotherapy regimen containing oxaliplatin (FOLFOX or FOLFOXIRI) prior to sampling. The average inhibition rate of oxaliplatin on hyperthermic perfusion of organoids was 37.7% (95% CI 24.7–50.8%) in the group with a history of oxaliplatin chemotherapy, and 38.0% (95% CI 30.2–45.8%) in the group without a history of oxaliplatin chemotherapy (independent sample t-test *p *= 0.972; the difference was not statistically significant). The average inhibition rate of oxaliplatin combined with 5-fluorouracil on the hyperthermic perfusion of organoids in vitro was 41.4% (95% CI 27.3–55.5%) in the group with a history of oxaliplatin chemotherapy, and 40.4% (95% CI 32.2–48.7%) in the group without a history of oxaliplatin chemotherapy (independent sample t-test *p *= 0.903; the difference was not statistically significant) (Fig. 3b–c). It has been determined that the previous administration of oxaliplatin chemotherapy does not influence the inhibition rate of oxaliplatin and oxaliplatin combined with 5-fluorouracil on hyperthermic perfusion of CRC organoids in vitro.

### RNA-Seq Analysis of Oxaliplatin Hyperperfusion Sensitivity

**Fig. 4:**
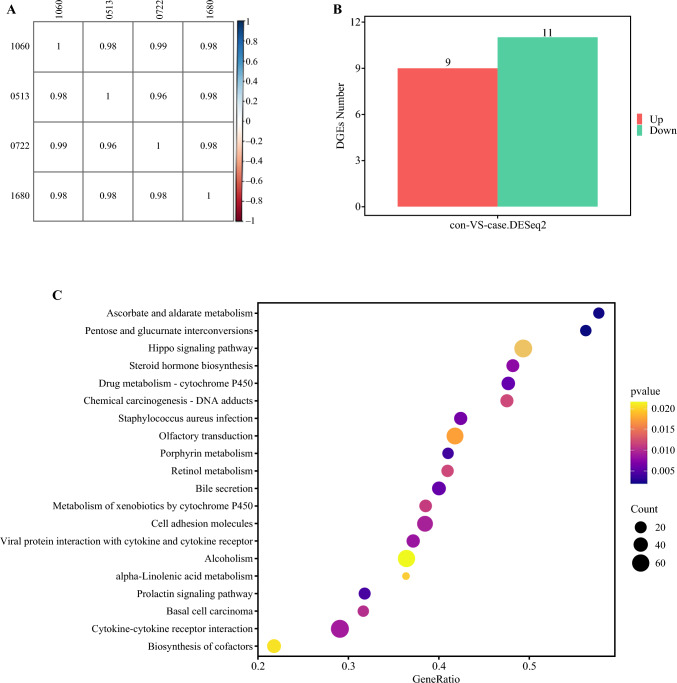
Transcriptome analysis. **A** Pearson correlation heat map. **B** Statistics of different expressio genes. **C** Pathway GSEA bubble plots for enrichment analysis. *GSEA* Gene Set Enrichment Analysis

Duplicate samples were chosen from two pairs of organoids, one sensitive and one insensitive to oxaliplatin, for the purpose of extracting total RNA for transcriptome sequencing. Construction of a transcriptome library was completed, followed by the implementation of quality control procedures for the raw data. Both the raw reads and clean reads of all samples exceeded 40 million. The Q30 value for each sample exceeded 91%, while the overall alignment rate of the genome surpassed 96% (Table 7). The Pearson correlation analysis revealed a correlation coefficient >0.95 between the samples, indicating excellent repeatability (Fig. 4a). This suggests that the sequencing data obtained is of high quality and is suitable for further analysis.

**Table 7:** Sample sequencing data

Sample number	Inhibition rate of oxaliplatin (%)	Raw reads	Clean reads	Q30 (%)	Total MAPPING ratio (%)
1060	61.8	48.80M	47.15M	91.34	96.57
0513	54.8	53.26M	51.07M	91.69	96.66
0722	0	51.53M	49.87M	91.44	96.49
1680	23.9	48.80M	42.42M	91.49	96.61

Our investigation utilizing differential gene expression analysis revealed a set of 20 genes potentially linked to oxaliplatin hyperthermic perfusion sensitivity (Fig. 4b), with 9 genes demonstrating significant upregulation in oxaliplatin-resistant organoids. A total of 11 genes exhibited downregulation, namely DEPP1, SYS1-DBNDD2, FAM106A, INO80B-WBP1, PRSS21, ACTL8, LGSN, IRX2, and CHMP1B2P, and including SLC26A3, CEACAM8, HMGCS2, NXPE4, SLC38A4, LINC00654, PRORP, PDE8B, ADAM2, SLC4A10, CA4. The KEGG enrichment analysis was performed on the transcriptome using the Gene Set Enrichment Analysis (GSEA) algorithm. The findings revealed a possible association between organoid resistance to oxaliplatin and multiple pathways, such as the Hippo signaling pathway, steroid hormone biosynthesis, drug metabolism − cytochrome P450, cell adhesion molecules, and retinol metabolism (Fig. 4c).

## Discussion

In this study, we successfully established an in vitro intraperitoneal hyperthermic perfusion system using organoids as a preclinical model, and evaluated the efficacy of four commonly used intraperitoneal hyperthermic perfusion chemotherapy regimens in vitro, achieving satisfactory results. HIPEC is used as a treatment for peritoneal tumors because it can directly expose tumor cells to chemotherapy drugs. The intraperitoneal route of administration offers significant concentration benefits compared with intravenous administration. The peritoneal plasma barrier, comprising the mesothelium and subcutaneous mesothelial tissue, results in significantly elevated drug concentrations within peritoneal tumor tissue compared with systemic administration.^30^ High concentrations of anticancer drugs can be in direct contact with tumor cells and can reduce the systemic concentration of chemotherapy drugs, thereby reducing systemic toxicity. Hyperthermia can enhance the penetration of chemotherapy drugs into tumor tissues and shows synergistic effects with a variety of anticancer drugs.^31^ However, the efficacy of HIPEC in the treatment of peritoneal tumors is controversial. The COLOPEC and PRODIGE7 studies have shown negative results and there is no consensus on the HIPEC regimen. It is difficult to design randomized controlled trials to verify the efficacy of different HIPEC regimens. Elias et al. conducted a two-center, prospective, randomized trial comparing intraperitoneal chemotherapy plus systemic chemotherapy with systemic chemotherapy alone from 1996 to 2000. In theory, although 90 patients were enrolled, in reality only 35 were enrolled, mainly because the trial was stopped early and the results were negative after patients who had been randomly assigned to systemic chemotherapy alone withdrew from the study.^32^ Therefore, it is more controllable and feasible to evaluate the effect of intraperitoneal hyperthermic perfusion chemotherapy in vitro than in clinical trials. Cancer cell lines, PDTXs, and organoids are commonly used in vitro models. At present, the research on the effect of HIPEC in vitro has involved three models. However, the generation of cancer cell lines from patient tumor tissue is very inefficient, involves the selection of two-dimensional (2D) culture conditions in vitro, and only rare clones are able to expand and maintain multiple passages. Derived cell lines may undergo major genetic changes and no longer reproduce the genetic heterogeneity and drug response of the original tumor.^14^ PDTX is a model constructed by transplanting fresh tumor tissue into immunodeficient mice. Klaver et al. used rats to demonstrate that cytoreductive surgery combined with HIPEC with mitomycin C in the treatment of colorectal peritoneal metastatic tumor-transplanted rats survived longer than with cytoreductive surgery alone.^33^ However, this method is expensive and time- and resource-consuming, and it is difficult to perform tumor cytoreduction surgery in rats, whereas organoid models ideally represent all cellular components of an organ,^34^ which can stably preserve the genetic, proteomic, morphological, and drug type characteristics of parental tumors in vitro. Furthermore, compared with PDTX cultures, the cycle is shorter and consumes less resources. HIPEC is based on complete cytoreductive surgery (tumor nodule diameter <2.5 mm), because the penetration of intraperitoneal chemotherapy to peritoneal carcinomatous or sarcoma nodules is limited to 1–2 mm,^35^ and heating can increase the penetration of drugs in specific sites to a depth of at least 5 mm.^36^ However, the size of the organoids used in the intraperitoneal hyperthermic perfusion experiment in this study is generally <0.1 mm. Therefore, the use of organoids as an in vitro model is more suitable for the detection of the efficacy of intraperitoneal hyperthermic perfusion chemotherapy. For the first time, Ubink et al. established an in vitro intraperitoneal hyperthermic chemotherapy model using five CRC PM tissue-derived organoids to evaluate the efficacy of oxaliplatin and mitomycin C, demonstrating that CRC PM-derived organoids can be used to evaluate existing intraperitoneal hyperthermic chemotherapy regimens at the individual patient level and to develop more effective treatment strategies.^37^ This study is the first to use CRC organoids to establish an organoid cohort of 42 patients to evaluate the efficacy of four intraperitoneal hyperthermic perfusion chemotherapy regimens, and statistically significant results were obtained.

The two current studies on organoids for hyperthermic intraperitoneal chemotherapy in vitro both tested the organoids for 3 days after hyperthermic perfusion of chemotherapy drugs,^37,38^ while organoids were generally used for drug sensitivity testing in vitro for no more than 6 days because the plates were easily affected by evaporation after the organoids were in contact with drugs for more than 6 days, which may result in decreased quality of the observed data.^39^ In this study, by detecting the inhibition rate at different time points after hyperthermic perfusion of mitomycin C in vitro, the best detection time for organoids after hyperthermic perfusion of drugs in vitro is 4–6 days, which is helpful for the promotion of this model.

According to the findings of this study, the average inhibition rate of mitomycin C on hyperthermic perfusion of CRC organoids was 85.2% (95% CI 80.4–89.9%). Mitomycin C was the inaugural drug employed for HIPEC, primarily targeting PM of gastric cancer. In their pioneering study, Fujimoto et al. administered mitomycin C and misonidazole for hyperthermic intraperitoneal chemotherapy in patients with gastric cancer and PM. The results clearly demonstrated the favorable tolerability of combining surgery with HIPEC using mitomycin C and misonidazole.^40^ In the 1990s, Witkamp et al. conducted a phase I/II trial to investigate the feasibility and effectiveness of cytoreductive surgery combined with intraoperative hyperthermic intraperitoneal chemotherapy with mitomycin C in patients with PM from CRC; 11/29 patients had surgical complications and one patient died as a direct result of the treatment. Mitomycin C toxicity was mainly manifested as leukopenia (52%). The 2- and 3-year survival rates were 45% and 23%, respectively.^41^ Mitomycin C is a cell cycle-specific drug that has a wide spectrum of anti-tumor activity. The fundamental mechanism of its anti-tumor effect involves depolymerizing cellular DNA and impeding DNA replication, thus suppressing the proliferation of tumor cells. It has been shown that heating can increase intraperitoneal MMC concentration with less systemic absorption.^42^ Cumulative dose-dependent myelosuppression, ranging from 28% to 83.6%, was identified as the most prevalent adverse effect of HIPEC.^43^ The reference dose of mitomycin C in this study was 35 mg/m^2^. Klaver et al. used rats for HIPEC and the results showed that the median survival time of rats treated with mitomycin C 15 and 35 mg/m^2^ was 75 and 97 days, respectively. However, the difference in survival between the two groups was not statistically significant (*p *= 0.197) and the toxicity of mitomycin C at the high dose (35 mg/m^2^) may offset its potential benefit.^33^ The dose-response curves of 9 organoids to mitomycin C at 37°C and 42°C in this study showed that heating had a sensitizing effect on low doses of mitomycin C, but the sensitizing effect on high doses of mitomycin C was not obvious. Moreover, high doses of mitomycin C had a high inhibition rate even without heating. Therefore, in order to reduce both the systemic absorption of mitomycin C and the incidence of adverse reactions, hyperthermia may not be used when using high-dose mitomycin C (35 mg/m^2^) in clinical practice. The American Society for Peritoneal Surface Malignancies consensus guideline on the standardization of HIPEC for CRC patients in the United States indicates that most surgical oncologists favor 90 min of HIPEC with mitomycin for patients undergoing cytoreductive surgery for colorectal peritoneal carcinomatosis.^44^ The results of the present study also showed a high inhibition rate of mitomycin C in vitro; therefore, mitomycin C can be used as the first choice for intraperitoneal hyperthermic perfusion chemotherapy.

In this study, the inhibition rate of small-dose mitomycin C (5 mg/L) combined with cisplatin in vitro hyperthermic perfusion was also very high, and the average inhibition rate reached 85.5% (95% CI 80.2–90.7%), which was similar to the effect of a large dose of mitomycin C alone. As an ancient anti-tumor drug, cisplatin can directly kill tumor cells in vitro and in vivo by causing intra- and inter-strand crosslinks and DNA-protein crosslinks,^45^ which provides a pharmacological basis for its use in HIPEC. As a hyperthermic intraperitoneal perfusion drug, cisplatin was mainly used in PM of gastric cancer and peritoneal mesothelioma, and was rarely used as a single drug in PM of CRC. A retrospective study in 2003 showed that 34 patients with PM from colorectal adenocarcinoma who underwent cytoreductive surgery combined with HIPEC with mitomycin C and cisplatin had a complication rate of 35%, a 2-year overall survival rate of 31%, a median survival time of 18 months, and a median time to local disease progression of 13 months.^46^ It has been reported that the incidence of grade 3–5 systemic adverse reactions of mitomycin C combined with cisplatin in HIPEC was 11.7%, and the main adverse reactions were myelosuppression and nephrotoxicity. The cisplatin dose >240 mg was an independent risk factor for grade 3–5 systemic adverse reactions.^47^ The cisplatin concentration used in this study was 20 mg/L, while the total amount was <240 mg, which is considered to be well tolerated. This dose was also confirmed in another study.^24^ Therefore, low-dose mitomycin C (5 mg/L) combined with a cisplatin regimen may have a lower incidence of adverse reactions than a high-dose mitomycin C (35 mg/m^2^) regimen, but the therapeutic effect is not inferior to high-dose mitomycin C 35 mg/m^2^.

5-fluorouracil is a cell cycle-specific drug that plays an anti-tumor role by inhibiting DNA synthesis and that has a good effect on digestive tract tumors. It is used for intraperitoneal chemotherapy in peritoneal malignancies because of its high peritoneal/plasma area under the curve ratio.^36^ Mitomycin C combined with 5-fluorouracil in HIPEC for CRC has good efficacy and safety in vitro and in vivo.^25^ In this study, the average inhibition rate of mitomycin C combined with 5-fluorouracil was 65.6% (95% CI 59.6–71.6%), which was weaker than that of mitomycin C alone and mitomycin C combined with cisplatin. Therefore, this regimen is not suitable as the first choice of intraperitoneal hyperthermic perfusion chemotherapy for PM from CRC.

The oxaliplatin regimen is the most controversial of this study’s results. In order to better mimic the clinical oxaliplatin HIPEC, we added a small dose of 5-fluorouracil to the clinical dose of oxaliplatin, but the results showed that neither oxaliplatin alone nor oxaliplatin combined with 5-fluorouracil had a very low inhibition rate on CRC organoids. The average inhibition rate of oxaliplatin was 37.9% (95% CI 31.5–44.3%) and the average inhibition rate of oxaliplatin combined with 5-fluorouracil was 40.7% (95% CI 33.9–47.5%); the difference was not statistically significant. Oxaliplatin belongs to the third-generation of platinum drugs and is the first platinum drug with an inhibitory effect on CRC.^48^ Oxaliplatin can not only be used as a HIPEC drug for CRC but also as a first-line systemic chemotherapy drug for CRC. Elias et al. conducted a phase II clinical study using oxaliplatin as a hyperthermic intraperitoneal perfusion drug in the late 20th century. The 3-year overall survival rates of 24 patients with CRC PM who underwent CRS+HIPEC were 83%, 74%, and 65%, respectively, and the 3-year disease-free survival rates were 70%, 50%, and 50%, respectively. Only 32% of the 22 patients who survived the surgery had peritoneal recurrence.^49^ This study laid the foundation for oxaliplatin as a HIPEC drug for CRC; however, the negative results of two recent clinical trials of HIPEC based on oxaliplatin (COLOPEC and PRODIGE7) have raised doubts about the efficacy of oxaliplatin. The results of in vitro sensitivity testing to oxaliplatin regimens in this study may, in part, explain the negative results of the two studies. One of the reasons for the low sensitivity of the oxaliplatin regimen may be the shorter duration of hyperthermic perfusion. An organoid model study showed that high-dose, short-course oxaliplatin (460 mg/m^2^, hyperthermic perfusion for 30 min) was not as effective as long-course, low-dose oxaliplatin (200 mg/m^2^, hyperthermic perfusion for 120 min), and that the inhibition rate of organoids in vitro was 53% and 31%, respectively.^38^ Another reason may be that the patient has a problem with resistance to oxaliplatin. Based on the results of the PRODIGE7 study, Sugarbaker believes that the use of oxaliplatin-containing chemotherapy before CRS+HIPEC may increase the resistance of tumor cells to oxaliplatin, therefore it is not suitable to use oxaliplatin as a HIPEC regimen.^50^ In the study of patients with CRC liver metastases, Andreou et al. showed that patients receiving oxaliplatin chemotherapy had a higher rate of RAS mutation, which was associated with poor prognosis after liver resection for metachronous CRC liver metastases.^51^ In this study, by comparing the inhibition rate of organoids with and without a history of oxaliplatin chemotherapy before tumor sampling, it was found that there was no statistical difference between the two groups. This suggests that there may be a subset of patients who are inherently insensitive to oxaliplatin.

There are many molecular mechanisms of oxaliplatin resistance.^52^ In this study, we performed transcriptome sequencing analysis of oxaliplatin hyperthermic perfusion-resistant and -sensitive organoids, and found that 9 genes were significantly upregulated and 11 genes were downregulated in oxaliplatin-resistant organoids. KEGG enrichment analysis showed that the resistance of organoids to oxaliplatin may be related to the Hippo signaling pathway, steroid hormone biosynthesis, drug metabolism − cytochrome P450, cell adhesion molecules, and retinol metabolism. Previous studies have shown that dysfunction of the Hippo pathway is associated with the development and metastasis of CRC.^53,54^ However, the expression of CYP1A2 and CYP2A6 was significantly increased in 5-fluorouracil- and oxaliplatin-resistant cells.^55^ Silencing of E-cadherin expression increases the chemosensitivity of CRC cell lines to irinotecan and oxaliplatin.^56^ Further studies are needed to confirm the mechanism of oxaliplatin resistance in CRC. Oxaliplatin may not be the first choice for HIPEC for CRC.

Our study has several limitations, including the low proportion of PM-derived organoids from CRC, because there are relatively few patients who undergo surgery for PMs and because samples from PMs tend to have more fibrotic tissue, which makes sampling and organoid culture less successful. To overcome this limitation, we compared the inhibition rates between PM tissue-derived organoids and primary CRC tissue-derived organoids using five in vitro hyperthermic perfusion protocols. The results showed that there was no significant difference in the inhibition rate of the two types of organs using the five in vitro hyperthermic perfusion protocols, indicating that the use of organoids cultured from primary colorectal tumor tissue can also simulate the intraperitoneal hyperthermic perfusion experiment, which is more helpful to the promotion of the model. Subsequently, peritoneal metastatic tissue samples can be added for further organoid culture and drug sensitivity testing. Second, the results of this study were based on an in vitro model experiment, and the drug concentration was calculated by combining the surface area of the mixture and the volume of the hyperthermic perfusion solution. Whether the drug concentration is similar to the clinical dosage needs further verification, therefore its guiding role in clinical decision making is limited.

## Conclusion

The in vitro hyperthermic perfusion of mitomycin C combined with cisplatin exhibits a higher inhibition rate compared with mitomycin C combined with 5-fluorouracil and oxaliplatin. In clinical practice, the combination of mitomycin C and cisplatin can be regarded as the primary option for intraperitoneal hyperthermic perfusion chemotherapy in patients with CRC.
